# Specific antibodies to *Anopheles* gSG6-P1 salivary peptide to assess early childhood exposure to malaria vector bites

**DOI:** 10.1186/s12936-015-0800-6

**Published:** 2015-07-22

**Authors:** Papa M Drame, Anne Poinsignon, Célia Dechavanne, Gilles Cottrell, Manon Farce, Rodolphe Ladekpo, Achille Massougbodji, Sylvie Cornélie, David Courtin, Florence Migot-Nabias, André Garcia, Franck Remoué

**Affiliations:** UMR MIVEGEC (IRD224-CNRS5290-Universités Montpellier 1 et 2), Institut de Recherche pour le Développement (IRD), BP64501, 34394 Montpellier, France; IRD-UMR MIVEGEC (IRD224-CNRS5290-Universités Montpellier 1 et 2), Centre de Recherche Entomologique de Cotonou (CREC), 01 BP 4414RP, Cotonou, Benin; IRD UMR 216 Mère et enfant face aux infections tropicales, 75006 Paris, France; Faculté de Pharmacie, Université Paris Descartes, Sorbonne Paris Cité, 75006 Paris, France; Laboratoire de Mathématiques Appliquées, Université Paris Descartes, 75006 Paris, France; Centre d’Etudes et de Recherche sur le Paludisme Associé à la Grossesse et à l’Enfant (CERPAGE), Cotonou, Benin; Faculté des Sciences de la Santé, Université d’Abomey-Calavi, 01 BP 188, Cotonou, Benin; Laboratory of Parasitic Diseases, NIAID, NIH, 4 Center Dr, Bethesda, MD 20892-0425 USA

**Keywords:** Malaria, Salivary proteins, Biomarker of exposure, *Anopheles* bites, Maternal IgG transfer, Infants, Africa

## Abstract

**Background:**

The estimates of risk of malaria in early childhood are imprecise given the current entomologic and parasitological tools. Thus, the utility of anti-*Anopheles* salivary gSG6-P1 peptide antibody responses in measuring exposure to *Anopheles* bites during early infancy has been assessed.

**Methods:**

Anti-gSG6-P1 IgG and IgM levels were evaluated in 133 infants (in Benin) at three (M3), six (M6), nine (M9) and 12 (M12) months of age. Specific IgG levels were also assessed in their respective umbilical cord blood (IUCB) and maternal blood (MPB).

**Results:**

At M3, 93.98 and 41.35% of infants had anti-gSG6-P1 IgG and IgM Ab, respectively. Specific median IgG and IgM levels gradually increased between M3 and M6 (p < 0.0001 and p < 0.001), M6–M9 (p < 0.0001 and p = 0.085) and M9–M12 (p = 0.002 and p = 0.03). These levels were positively associated with the *Plasmodium falciparum* infection intensity (p = 0.006 and 0.003), and inversely with the use of insecticide-treated bed nets (p = 0.003 and 0.3). Levels of specific IgG in the MPB were positively correlated to those in the IUCB (R = 0.73; p < 0.0001) and those at M3 (R = 0.34; p < 0.0001).

**Conclusion:**

The exposure level to *Anopheles* bites, and then the risk of malaria infection, can be evaluated in young infants by assessing anti-gSG6-P1 IgM and IgG responses before and after 6-months of age, respectively. This tool can be useful in epidemiological evaluation and surveillance of malaria risk during the first year of life.

## Background

In the absence of effective vaccines, malaria control and prevention strategies are primarily based on the widespread use of effective drugs and vector control by insecticide-treated bed nets (ITNs) and indoor residual sprays [1–3]. Such interventions are associated with recent decline in malaria burden across a range of settings [4–6]. However, malaria incidence and risk are static or increasing in several African countries, including the Republic of Benin, where the disease is still the first leading cause of child morbidity and mortality [7].

Naïve newborns are at increased risk of malaria infection during the first year of life in endemic areas [8–10]. Newborn exposure to malaria has traditionally been assessed using entomological and parasitological methods. However, these methods are labour-intensive and difficult to sustain especially in low transmission or exposure contexts [11, 12]. In addition, these methods are typically used at the community level measure and do not target the individual [13]. Moreover, these methods typically exclude infants or newborns because of numerous ethical concerns [14, 15]. Evaluating the risk of malaria in vulnerable infants is not only challenging but also requires new tools [16, 17].

Improving an understanding of human-*Anopheles* interactions can potentially provide a promising alternative [18, 19]. *Plasmodium* spp. are transmitted to humans by the saliva of infected-female *Anopheles* mosquitoes during a blood meal. Injected mosquito salivary molecules facilitate the mosquito blood-feeding activity both by counteracting the human haemostatic and inflammatory reactions and by modulating its innate and adaptive immune responses [20, 21]. Following mosquito infecting as well as not-infecting bites, humans produce immunoglobulin G (IgG), M (IgM), and/or E (IgE) specific to injected mosquito salivary proteins [22–24]. Such humoral responses may be a marker of human exposure to vector bites and pathogens associated with mosquito-borne diseases [25–30].

A specific and highly conserved *Anopheles gambiae* salivary gland protein-6 peptide 1 (gSG6-P1) antigen has been validated as a biomarker of *Anopheles* bites [13]. The anti-gSG6-P1 IgG response has been associated with local exposure level to *An. gambiae s.l.* and *Anopheles funestus s.s.* bites in zones with various transmission intensities [22, 31–33]. Moreover, anti-gSG6-P1 IgG responses have been shown to reflect the success of ITN-based [31, 34] and other [34] malaria vector control methods in that they diminish quite rapidly when exposure level drops [31]. However, this tool has not yet been tested in vulnerable young infants (<1-year old), therefore limiting its field application to malaria epidemiological studies or surveys.

The present study aimed to evaluate and to follow, during the first months of life, the acquisition level of human IgM and IgG responses to gSG6-P1 in southern Benin. Associations between specific gSG6-P1 IgG and IgM responses and parasitological and entomological parameters were evaluated. Whether mothers provide their infant with specific IgG through fetal-maternal transfer was also investigated.

## Methods

### Study area

The study was conducted in the district of Tori Bossito, a rural area located on the coastal plain of south Benin, 40 km northwest of Cotonou (the economic capital city). It is a sub-tropical area with an annual average rainfall of 1,100 mm. There are two rainy seasons: April–July and October–November. The distribution of rainfalls, maximal during the two rainy seasons, was heterogeneous over the area. Average temperatures monthly varied between 27 and 31°C. *Anopheles gambiae s.s.* and *An. funestus,* the most abundant anopheline species, are the major vectors for the transmission of *Plasmodium falciparum*, the major human malaria parasite in this region [35, 36]. A recent study has described an average annual *P. falciparum* entomological inoculation rate of 15.5, with important time and space variations depending on villages [37]. *Anopheles nili*, *Anopheles pharoensis* and *Anopheles leesoni,* also locally present, are not yet associated with malaria transmission [36].

### Study design, population and sampling

The study included nine villages (Avame Centre, Gbedjougo, Houngo, Anavie, Dohinoko, Gbetaga, Tori Cada Centre, Zebe, and Zoungoudo) distributed within three maternity hospitals (MHs: Avame, Cada and Gare) providing birth attendance and primary health care. Women who attended the maternity hospitals for antenatal care were asked to enter into the study and were recruited from anytime after the 7th month of pregnancy. Inclusion criteria were to: (1) live in one of the nine villages; (2) have no intention to move within the next 12 months; and, (3) plan to deliver at the maternity hospital. At delivery, maternal peripheral (MPB) and infant umbilical cord (IUCB) blood spots were collected on filter papers (Whatman No 1, Saint-Louis, MO, USA). After delivery, newborns were actively followed monthly over the subsequent 12 months. Capillary blood on filter paper and venous blood for smears (thick blood smears) were collected every 3 months (M3, M6, M9, and M12) for each live newborn to study the kinetics of antibody responses to gSG6-P1 peptide and to determine the presence and/or intensity of *P. falciparum* infections. Parasite density (parasitaemia) was calculated as the number of *P. falciparum* (99% of identified *Plasmodium*) parasites per microlitre of blood; mean parasitaemia values (*x* + 1) were calculated. Mothers were asked to fill out questionnaires, including the use of ITNs and any clinical sign of the malaria for themselves and their babies. A birth cohort was then set up in July 2007 and recruitment performed until July 2008 as previously described [37].

The IRD Consultative Committee on Professional Conduct and Ethics (5 May, 2008) and the Ethical Committee of the Faculté des Sciences de la Santé of the University of Abomey-Calavi in Benin approved the study. All necessary permits were obtained for the described field studies. Informed written consent in French and Fon (major spoken language in the studied region) was obtained from the mothers.

Immunoassays were performed for 133 infants who completed a cord blood and the all four three-monthly blood spot samplings during the first 12 months of life follow-up.

### Prediction of spatiotemporal risk of malaria transmission

Entomological data (by human landing catch of mosquitoes) were collected every 6 weeks on three successive nights from July 2007 to July 2008 at four catch houses (four indoors and four outdoors) in each village, as previously described [38]. A statistical model predicting the spatiotemporal transmission of malaria at the household level in all nine villages was then developed based on correlation results between *Anopheles* density and climatic/environmental factors [38], allowing comparisons between the individual risk of malaria transmission data from that model to anti-gSG6-P1 IgG and IgM Ab levels.

### Salivary peptide gSG6-P1

The gSG6-P1 peptide was designed using bioinformatics to maximize its *Anopheles* specificity, synthesized and purified by Genepep SA (Saint Jean de Vedas, Herault, France). It was used for immunological tests (ELISA) as previously described [13, 32].

### Human IgG and IgM assays by indirect ELISA

Standardized dried blood spots were eluted as previously described [28]. ELISAs were carried out on blood spot eluates to assay IgG and IgM responses to gSG6-P1. Briefly, Maxisorp plates (Nunc, Roskilde, Denmark) were coated with gSG6-P1 antigen (20 μg/mL) in PBS. Plates were blocked using 300 µL of Protein-Free Blocking-Buffer (Pierce, Thermo Scientific, France) for 45 min at 37°C. Eluates were diluted 1/40 (IgG) or 1/20 (IgM) in PBS-Tween-1% and incubated in duplicate at 4°C overnight. Monoclonal mouse biotinylated Ab against human IgG or IgM (BD Pharmingen, San Diego, CA, USA) were incubated at 1/2,000 (IgG) or 1/500 (IgM) dilution. Peroxidase-conjugated streptavidin (Amersham, Les Ulis, France) was then added (1/2,000 for IgG and 1/500 for IgM). Colorimetric development was carried out using ABTS (2,2’-azino-bis (3-ethylbenzothiazoline-6-sulfonic acid) diammonium; Sigma, St Louis, MO, USA) in 50 mM citrate buffer (pH 4) containing 0.003% H_2_O_2_. The optical density (OD) was measured at 405 nm. In parallel, each test sample was assessed in a blank well containing no gSG6-P1 antigen (ODn). Results were expressed as the ΔOD value: ΔOD = ODx − ODn, where ODx represents the mean of individual OD in both antigen wells. Anti-gSG6-P1 IgG and IgM levels were also assessed in 14 non-*Anopheles* exposed individuals (negative controls) from northern France to calculate the immune response threshold (TR). The TR was calculated by the following formula: TR = mean (∆OD_neg_) + 3SD = for IgG for IgM. In the present study an IgG responder had a ΔOD_IgG_ > 0.204 and an IgM responder had a ΔOD_IgM_ > 0.288.

### Statistical analysis

Data were analysed using R (version 2.14.1), and graphs were constructed with GraphPad Prism5^®^ software (San Diego, CA, USA). An simple linear mixed effect (LME) regression model was constructed to determine the correlation between anti-gSG6-P1 IgG or IgM response and each of potential epidemiological, biological and environmental factors, such as age (M3, M6, M9, and M12), gender (male *vs* female), the season of sampling (end dry season: February to April; beginning rainy season: May to July; end rainy season: August to October; beginning dry season: November to January), *P. falciparum* prevalence (yes/no), *P. falciparum* infection intensity (continuous variable), bed net use (yes/no), and environmental risk of exposure (continuous variable). Then, a multivariate LME analysis was performed by including all explanatory variables that have shown significant (or close) *p* value in the univariate (simple) model. In this multivariate model, two random intercepts at individual and village levels were introduced, correcting then biological inter-individual and inter-village variations. Two or more percentages of immune responders were compared using the Fisher’s exact-test. Spearman rank correlations were used to estimate the force of the association between specific IgG in the MPB, IUCB and M3-infants’ peripheral (M3) bloods.

## Results

### Evolution of specific IgG and IgM levels during the first year of life

**Figure 1:**
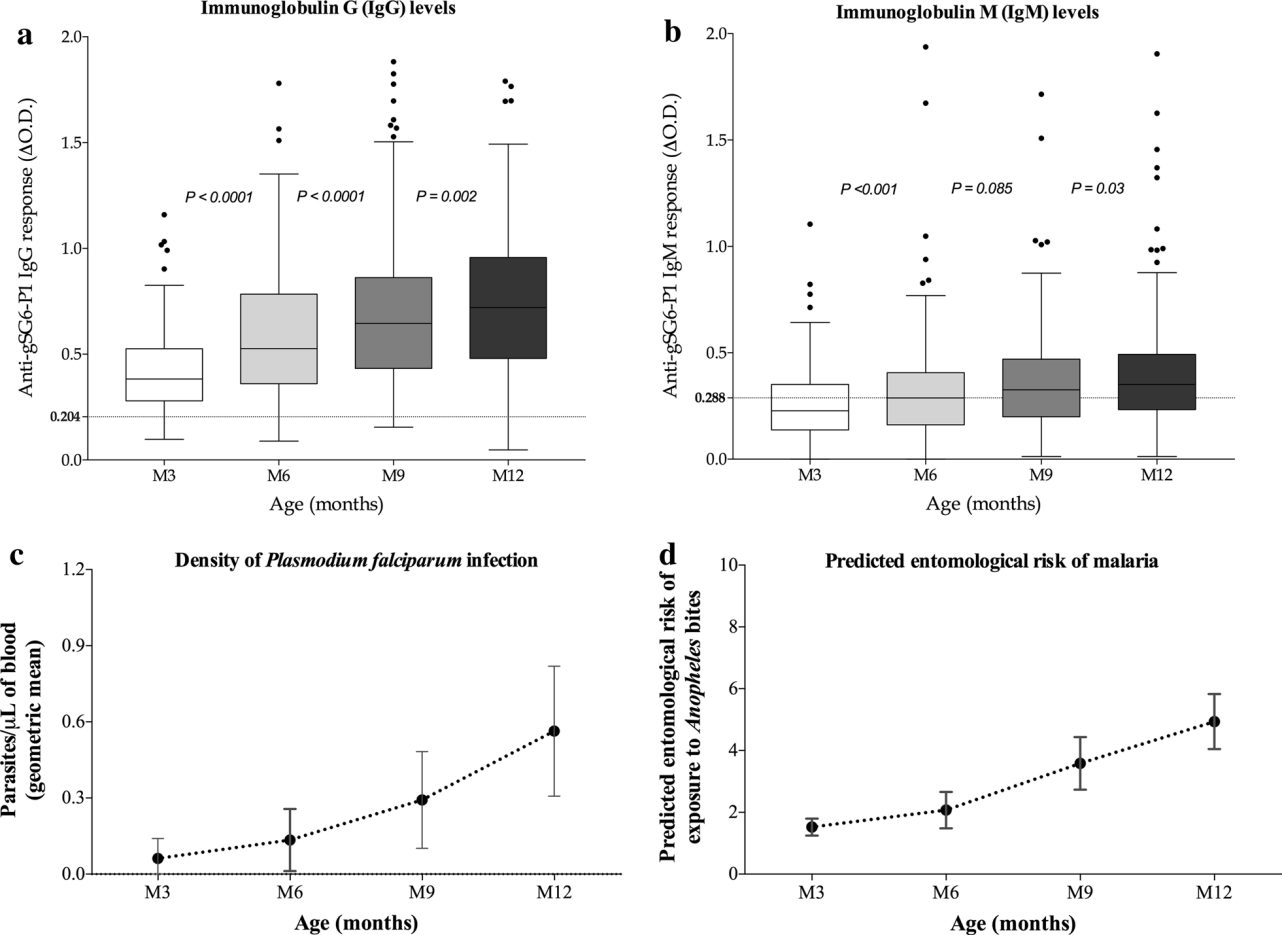
IgG and IgM responses to gSG6-P1 salivary peptide, intensity of *Plasmodium falciparum* transmission and *Anopheles* exposure level in the first year of life. Individual IgG (1a) and IgM (1b) responses to the *Anopheles* gSG6-P1 peptide biomarker are represented for infants in months 3 (*white*), 6 (*light-grey*), 9 (*dark-grey*) and 12 (*black box*) after their birth. *Boxes* locate the middle 50% of the data; *horizontal lines* in the *boxes* indicate median values; lengths of boxes correspond to the inter-quartile ranges. *Horizontal black dotted lines* represent the cut-off of IgG (0.204) and IgM (0.288) responder. The intensity of *P. falciparum* infection (1c) and the entomological risk of malaria (1d) are also represented for the same four age-sampling periods. *Vertical lines* indicate confidence intervals of the mean values. Statistical significant differences between all age groups (multivariate linear mixed model analysis) are indicated.

At month 3 (M3), levels of specific gSG6-P1 IgG (Figure 1a) and IgM (Figure 1b) in the peripheral blood of infants were low [median IgG level (M_IgG_) = 0.383 and median IgM level (M_IgM_) = 0.228]. Importantly, these levels of IgG and IgM gradually increased from M3 to M12 (M_IgG_ = 0.721 and M_IgM_ = 0.352), with intermediate values at M6 (M_IgG_ = 0.527 and M_IgM_ = 0.288) and M9 (M_IgG_ = 0.646 and M_IgM_ = 0.326). The increase of specific IgG levels (Figure 1a) was significant between two successive sampled age periods: M3–M6 (p < 0.0001), M6–M9 (p < 0.0001) and M9–M12 (p = 0.002). Similarly, specific IgM levels (Figure 1b) significantly increased only between M3 and M6 (p < 0.001) and M9–M12 (p = 0.03). No significant increase in specific IgM levels was noted between M6 and M9 (p = 0.085).

Interestingly, as observed for anti-gSG6-P1 IgG and IgM, the intensity of *P. falciparum* infection in infants (Figure 1c) and the predicted entomological risk of infant exposure to *Anopheles* bites (Figure 1d) were also low at M3 and increased from M3 to M12. However, the difference in the intensity of *P. falciparum* infection and the predicted entomological risk of exposure between any two consecutive age periods (M3–M6, M6–M9 and M9–M12) were not significant, unlike that observed for anti-gSG6-P1 Ab levels.

The percentages of IgG and IgM responders were calculated for the four time-sampling dates (Table 1). Results show that by M3, 94% of the infants were IgG responders, and this increased to 98% by M6 and to 99% by M9–M12. The percentage of IgM responders was lower at M3 (41%), and gradually increased to 65% by M12 with values of 50% at M6 and 56% at M9.

**Table 1:** Percentages of IgG and IgM responders according to age and the place of birth/residence

Variables	Groups	Individual Ab responders to gSG6-P1 peptide	
		Percentage (%) of IgG responders	Percentage (%) of IgM responders
Age	M3	93.98 (125/133)	41.35 (55/133)
	M6	97.74 (130/133)	50.37 (67/133)
	M9	98.96 (131/133)	56.39 (75/133)
	M12	98.96 (131/133)	65.41 (87/133)
Maternity hospitals	Avame	97.87 (230/235)	17.02 (40/235)
	Gare	93.11 (108/116)	89.65 (104/116)
	Cada	98.37 (181/184)	76.63 (141/184)

The place of birth/residence was studied by combining infants of all ages (n = 532).

### Specific gSG6-IgG levels according to maternity hospital of delivery

**Figure 2:**
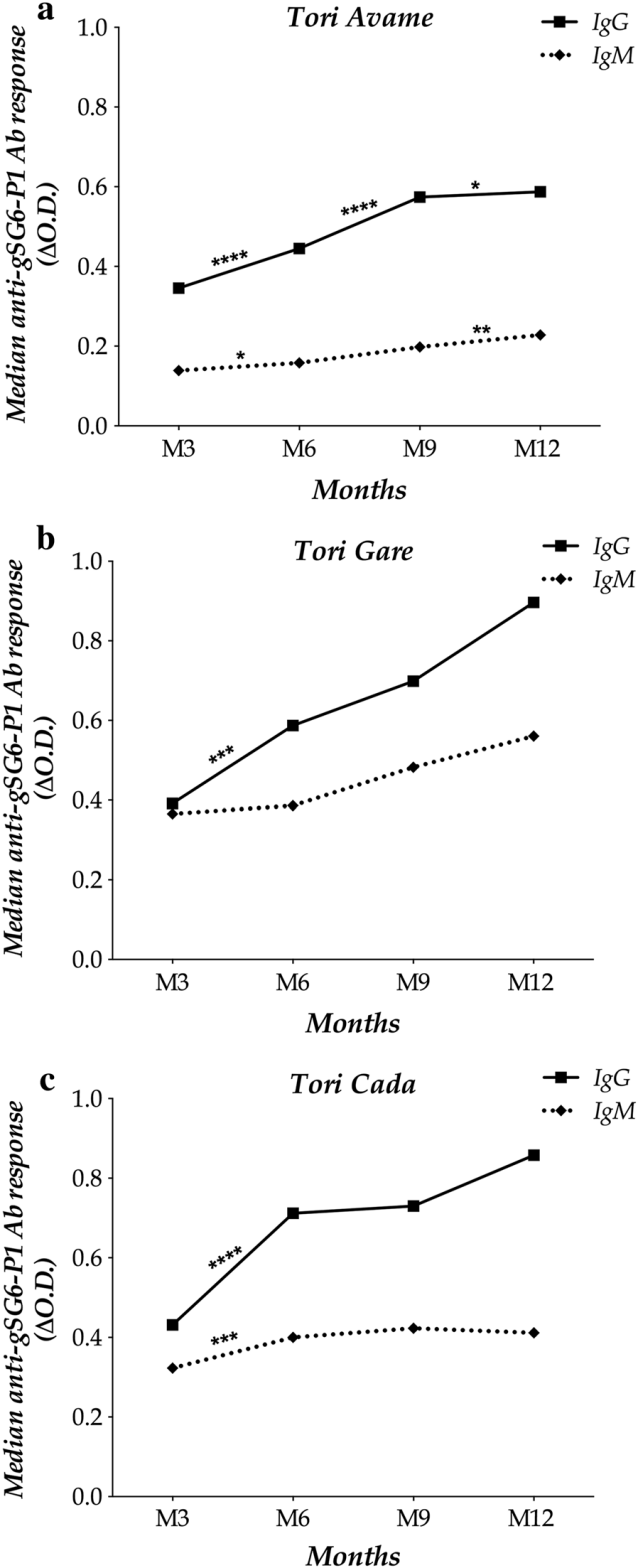
Evolution of gSG6-P1-specific IgG and IgM in the maternity hospital of infants’ birth. The evolution of IgG (*solid line*) and IgM (*dotted line*) responses to gSG6-P1 *Anopheles* antigen is represented for infants in Tori Avame (**a**), Tori Gare (**b**) and Tori Cada (**c**). The significance level of the difference of specific Ab level between two successive age-periods is indicated by “*” (“*”, “**” and “***” mean p < 0.05, p < 0.001 and p < 0.0001, respectively).

Assuming that each pregnant woman delivered her baby at the closest maternity hospital, Immunological data were analysed based on the hospital of delivery. Avame, Gare and Cada received pregnant women from three (Avame centre, Gbedjougo and Houngo), two (Dohinoko and Gbetaga) and four villages (Anavie, Cada, Zebe and Zoungoudo), respectively. The M3 levels of IgG and IgM (Figure 2) and the percentage of IgM responders at M3 (Table 1) were lower in Avame compared to Gare (p = 0.0013, p < 0.0001 and p < 0.0001, respectively) or to Cada (p < 0.0001 for all three), although the percentage of IgG responders (Table 1) was not significantly different among the three locations of birth. Median IgG levels did not differ between Gare and Cada (p = 0.47), in contrast to the median IgM levels, which were higher in Gare (p = 0.0008).

The profile of median of specific Ab levels during the first life year was similar in the three MHs: a gradual increase from M3 to M12 (Figure 2). However, some differences between two successive age-sampling periods were observed. In detail, in Avame (Figure 2a) where anti-gSG6-P1 Ab responses were lower, IgG levels increased significantly between M3 and M6 (p < 0.0001), M6–M9 (p < 0.0001), M9–M12 (p < 0.05), and IgM levels between M3 and M6 (p < 0.05), M9–M12 (p < 0.01). However, IgG and IgM levels significantly increased only between M3 and M6 in Gare (p < 0.001 for IgG; Figure 2b) and in Cada (p < 0.0001 and p < 0.001 respectively; Figure 2c).

### Age, intensity of *Plasmodium falciparum* infection, use of vector control and exposure to *Anopheles* bites: main factors of variation of anti-gSG6-P1 IgG and IgM levels

Independent of location and individual cofactors (random intercepts at individual and village levels), both individual IgG and IgM responses to gSG6-P1 were 20 and 8% higher at M6 (regression coefficient (RE) = 1.20 and p < 0.0001, RE = 1.08 and p = 0.0117, respectively), 34 and 12% at M9 (RE = 1.34 and p < 0.0001, RE = 1.12 and p < 0.0001, respectively) and 39 and 16% at M12 (RE = 1.39 and p < 0.0001, RE = 1.16 and p < 0.0001, respectively) compared to M3 (Table 2). Specific IgG and IgM levels were also positively associated with the density of *P. falciparum* infection (RE = 1.03 and p = 0.0065, RE = 1.03 and p = 0.0037, respectively). In contrast, anti-gSG6-P1 IgG responses significantly decreased with the use of vector control (ITNs) (RE = 0.47 and p = 0.0037). Finally, no significant variation of specific gSG6-P1 IgG or IgM levels was observed according to seasonality, the predictive risk of exposure to malaria or the prevalence of infection with *P. falciparum* (Table 2).

**Table 2:** Linear Mixed-Effects (LME) model to study anti-gSG6-P1 response according to age, gender, seasonality, vector control, location and *Plasmodium falciparum* infection

	Anti-gSG6-P1 IgG response		Anti-gSG6-P1 IgM response	
	Estimated coefficient (SE)	P-value	Estimated coefficient (SE)	P-value
Fixed effects				
Intercept	*0.57* (0.09)	*0.0000*	*0.36* (0.06)	*0.0000*
Age (months)				
3 (n = 133)	–	–	–	–
6 (n = 133)	*0.184* (0.024)	*0.0000*	*0.081* (0.025)	0.0117
9 (n = 133)	*0.289* (0.025)	*0.0000*	*0.110* (0.026)	0.0000
12 (n = 133)	*0.327* (0.026)	*0.0000*	*0.152* (0.026)	0.0000
Season				
Dry season 1	–	–	–	–
Dry season 2	0.57 (0.09)	0.1445	-0.001 (0.028)	0.9561
Rain season 1	0.57 (0.09)	0.3689	-0.040 (0.044)	0.3590
Rain season 2	0.57 (0.09)	0.7927	-0.029 (0.030)	0.3218
Vector control				
No ITN	–	–	–	–
ITN	−*0.075* (0.025)	*0.0037*	−0.023 (0.027)	0.3904
*Plasmodium* prevalence				
Not infected	–	–	–	–
Infected	0.016 (0.064)	0.8084	0.089 (0.068)	0.1943
*Plasmodium* density	*0.031* (0.011)	*0.0065*	*0.031* (0.010)	*0.0037*
Entomological risk of exposure	−0.003 (0.004)	0.4677	*0.009* (0.005)	*0.0578*
Random effects				
Village level	0.074 (0.045)	–	0.091 (0.086)	–
Individual level	0.241(0.183)	–	0.157 (0.202)	–

The classes of variables with significant *p* value are highlighted in italic

The intercept is the constant value when all independent variables are zeros (e.g. the value of median Ab response in someone with no risk factors). Random effects of the model were estimated at individual and village levels in the form of standard deviation. The estimated coefficient, its confidence interval and the degree of signification (P-value) are indicated. A positive regression coefficient means that the explanatory variable increases the probability of Ab response to gSG6-P1, while a negative regression coefficient means the contrary.

### Evidence of mother-to-child transmission of anti-gSG6-P1 IgG

**Figure 3:**
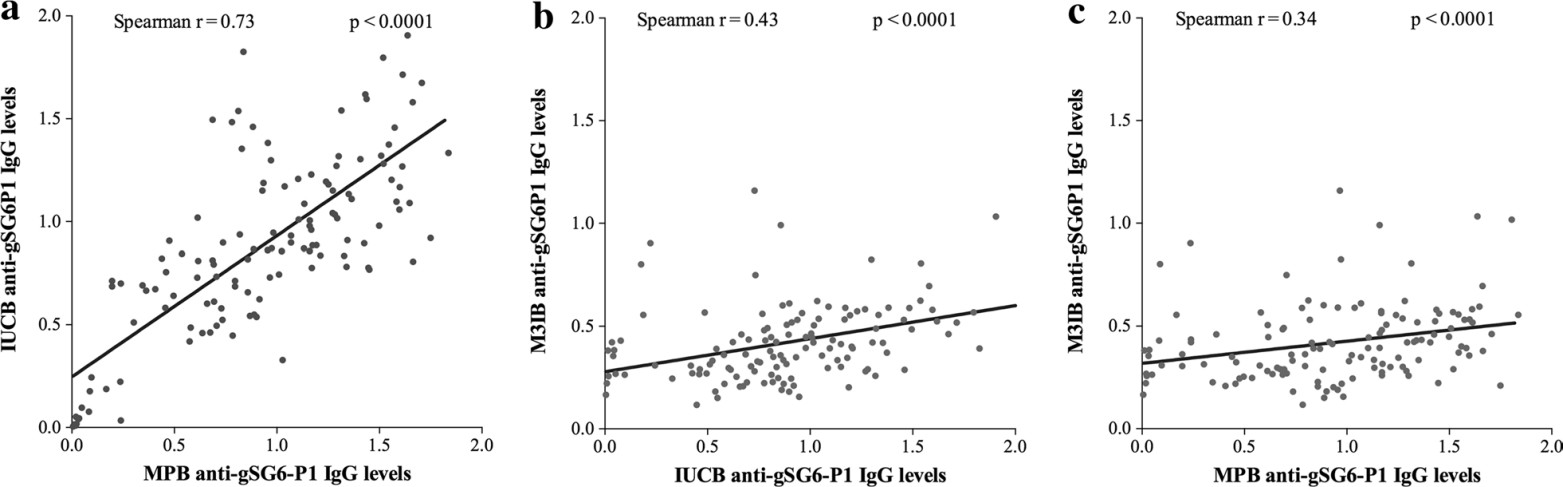
Correlations between specific IgG levels in the mothers’ peripheral blood, umbilical cord blood and blood of M3-infants. Anti-gSG6-P1 IgG individual levels (*black points*) in the mothers’ peripheral blood (MPB) are correlated to those detected in the infants’ umbilical cord (IUCB; Figure 2a) and in the M3-infants’ blood (Figure 2c). Specific IgG in the IUCB are correlated to those in M3-infant blood (Figure 2b). The *black solid line* indicates the correlation line. The significance of the linear association between two compared parameters is indicated (Spearman correlation method).

As mentioned before, the percentage of specific IgG responders was surprisingly high (93.98%), and considerably higher (p < 0.0001) than that of anti-gSG6-P1 IgM responders (41.35%). In addition, a positive association was observed between specific IgG levels in the MPB and the IUCB (Spearman R = 0.73; p < 0.0001; Figure 3a), in the IUCB and the peripheral blood of M3-infants (R = 0.43; p < 0.0001; Figure 3b), and in the MPB and the peripheral blood of M3-infants (R = 0.34; p < 0.0001; Figure 3c). Moreover, a positive association was observed between specific IgG levels in the MPB and the M3-infant bloods in Avame (Spearman R = 0.36; p = 0.006) and Cada (R = 0.40; p = 0.005), and not in Gare (R = 0.07; p = 0.7).

## Discussion

This study reports the presence of IgG and IgM Ab specific to gSG6-P1, a salivary biomarker of *Anopheles* bites, in the blood of Beninese infants. A major point is that levels of such IgG and IgM responses gradually increased from three to 12 months of age, in parallel with the intensity of infection with *P. falciparum* and the predicted entomological risk of exposure to *Anopheles* bites. These data extend to infants the utility of such a biomarker and show also that *mother*-*to*-*child* transplacental transfer of IgG specific to a salivary protein of a blood-feeding arthropod occurs. This transfer, however, could cause an overestimate of the risk of malaria exposure or transmission during early childhood in a high endemic malaria area.

In the study, anti-gSG6-P1 IgG and IgM Ab were detected in respectively 93.98 and 41.35% of infants at 3 months (M3) of age. *Anopheles* mosquitoes then early bite the majority of infants in these localities of south Benin. However, the pressure of mosquito bites they were exposed to at 3 months was not high, as pointed by the low median values of IgG (M = 0.383, slightly upper the ‘TR_IgG_’) and IgM (M = 0.228, under the TR_IgM_). These ‘low’ endogenous M3-levels of plasma specific antibodies were not surprising, in that the newborn immune system is relatively considered as immature during early infancy [39–41]. In endemic malaria areas, young infants are progressively exposed to *Anopheles* bites, their salivary antigens, and eventually the *Plasmodium* spp. they carry [42]. This is suggested by the correlation observed between anti-gSG6-P1 Ab and both the intensity of infection with *P. falciparum* and the entomological risk of infant exposure to *Anopheles* bites [22, 26, 28, 33].

Specific IgG and IgM levels significantly differed based on the location of residence, suggesting spatial differences in human-*Anopheles* contact levels. Infants in the Avame area had lower specific IgG and IgM responses and presumably a lower contact level with *Anopheles* during their first year of life. This can be linked to a likely lower aggressiveness of *Anopheles* in this area, a higher protection level against vectors or different household characteristics (type, well-closed doors and windows) [22, 31–33, 38]. This might explain the slower gradual increase of specific IgG and IgM in Avame compared to Cada and Gare. The role of individual factors (genetic background, nutritional status or nocturnal activities) in that differential gradual acquisition cannot be excluded [37, 43, 44]. All this means that infants were more highly bitten by mosquitoes, acquired more rapidly maturity of immune system and synthetized more specific antibodies in Gare settings than in Avame and Cada.

Maternal IgG is provided to the infant by transplacental transport [9, 45]. This study describes the evidence of a mother-infant transplacental transfer of anti-gSG6-P1 IgG, contributing to the high percentage of IgG infant responders at 3 months of age (about 94%). Maternal blood concentrations of specific IgG were positively correlated with respective umbilical cord and three-month old infant blood values. In addition, higher specific IgG levels were found in the maternal and cord blood of M3-infants with higher IgG titers, suggesting that the quantity of transferred specific IgG is dependent on the level in the mother that likely reflects the level of maternal exposure to *Anopheles* bites. Despite this positive association, maternal levels of specific IgG transfer could not accurately predict the level of infant specific IgG. In addition, such transfer also depends on the placental integrity, IgG subclass and nature of antigen [9, 46]. Unfortunately, investigating the effects of such factors was not possible in this study.

For a reliable evaluation of the young infant-*Anopheles* contact level by using anti-gSG6-P1 IgG, the proportion contributed from the mother must be considered. The linear regression analysis has estimated about 52 and 21% of the maternal gSG6-P1-specific IgG found in blood of newborns just after delivery and at 3 months of life, respectively, indicating a rapid decline of maternally transferred IgG [47, 48]. It also suggests that maternally transferred IgG are probably cleared in the blood of infants by 6 months of age, suggesting that anti-gSG6-P1 IgG should be more relevant as biomarker of infant exposure to mosquito bites after 6 months of age. Another possibility would be to distinguish maternal and neonatal specific Ab in infant plasma by mass spectrometry [49], but such technology is unavailable in resource-limited countries. The third approach would be to use IgM Ab that form polymers (usually pentamers) in humans and does not cross the maternal-feta barrier [9, 50]. Interestingly, the M3 proportion of infants presenting IgM against gSG6-P1 peptide (41.35%) was significantly lower than the percentage of those with specific IgG (93.98%). In addition, the highest percentage of IgM responders (89.65%) was obtained in Gare areas, where levels of mother specific IgG were not correlated to levels of IgG of their M3-infants, contrary to Avame and Cada areas. Moreover, no differences in IgM levels or percentage of responders were observed between two successive sampling ages after 6 months of age, in contrast to IgG levels. In infants older than 6 months, specific IgG levels were better at assessing exposure to *Anopheles* bites and the risk of malaria transmission. One hypothesis is that, depending on the level of exposure to mosquito bites, the initial anti-*Anopheles* saliva IgM response may have failed to switch to IgG after several weeks or months as has been shown previously for responses to *P. falciparum* antigens [50, 51]. Nevertheless, the kinetics of IgG (and subclasses) and IgM responses to gSG6-P1 salivary peptide must be elucidated in additional studies.

## Conclusion

In summary, individual exposure levels to *Anopheles* mosquito bites and therefore the risk for malaria transmission in early infancy has been described in a malaria-endemic area of Benin. Moreover, because of transfer of IgG specific to salivary gSG6-P1 *Anopheles* peptide from mother to infant there may be an overestimation of the utility of such a ‘biomarker’ early in infancy (less than 3 months). To overcome that potential challenge, specific IgM and IgG isotypes may be assayed for in infants younger and older than 6 months, respectively.
